# Small ruminant lentivirus genetic subgroups associate with sheep *TMEM154* genotypes

**DOI:** 10.1186/1297-9716-44-64

**Published:** 2013-07-29

**Authors:** Lucia H Sider, Michael P Heaton, Carol G Chitko-McKown, Greg P Harhay, Timothy PL Smith, Kreg A Leymaster, William W Laegreid, Michael L Clawson

**Affiliations:** 1 United States Department of Agriculture (USDA) Agricultural Research Service (ARS), U.S. Meat Animal Research Center (USMARC), State Spur 18D, Clay Center, NE 68933, USA; 2 Present address: Embrapa Caprinos e Ovinos, Estrada Sobral-Groaíras, km 4, Sobral CE, 62.010-970, Brazil; 3 Department of Veterinary Sciences, University of Wyoming, 1174 Snowy Range Road, Laramie, WY 82070, USA

## Abstract

Small ruminant lentiviruses (SRLVs) are prevalent in North American sheep and a major cause of production losses for the U.S. sheep industry. Sheep susceptibility to SRLV infection is influenced by genetic variation within the ovine transmembrane 154 gene (*TMEM154*). Animals with either of two distinct *TMEM154* haplotypes that both encode glutamate at position 35 of the protein (E35) are at greater risk of SRLV infection than those homozygous with a lysine (K35) haplotype. Prior to this study, it was unknown if *TMEM154* associations with infection are influenced by SRLV genetic subgroups. Accordingly, our goals were to characterize SRLVs naturally infecting sheep from a diverse U.S. Midwestern flock and test them for associations with *TMEM154* E35K genotypes. Two regions of the SRLV genome were targeted for proviral amplification, cloning, sequence analysis, and association testing with *TMEM154* E35K genotypes: *gag* and the transmembrane region of *env*. Independent analyses of *gag* and *env* sequences showed that they clustered in two subgroups (1 and 2), they were distinct from SRLV subtypes originating from Europe, and that subgroup 1 associated with hemizygous and homozygous *TMEM154* K35 genotypes and subgroup 2 with hemi- and homozygous E35 genotypes (*gag p* < 0.001, *env p* = 0.01). These results indicate that SRLVs in the U.S. have adapted to infect sheep with specific *TMEM154* E35K genotypes. Consequently, both host and SRLV genotypes affect the relative risk of SRLV infection in sheep.

## Introduction

Small Ruminant Lentiviruses (SRLVs) are heterogeneous slow-growing RNA viruses within the Retroviridae family that infect domestic sheep, goats, and some wild ruminants [1-6]. SRLVs have a primary tropism for monocytes, macrophages, and dendritic cells, and employ a “Trojan Horse” mechanism to disseminate in an immunocompetent host, whereby they infect circulating monocytes and remain quiescent until the monocytes mature into macrophages and become tissue-activated [7-9]. There are no known cures or efficacious vaccines for SRLVs [10-12]. Once established, SRLV infections persist throughout the lifetime of the host, and typically result in a short, acute disease episode that resolves into a protracted incubation period and slow, variable progression to disease [1,3,13]. Sheep under two years of age rarely show signs of disease. However, some may be infected ten years before displaying clinical symptoms, while other infected animals never display clinical symptoms [7,13].

SRLV-induced disease results from chronic inflammation [6,9,11]. In sheep, common symptoms include interstitial lung pneumonia with accompanying dyspnea, indurative mastitis, and cachexia, whereas ataxia and arthritis occur more rarely [3,13,14]. With exceptions that include Australia, Iceland, and New Zealand, SRLVs are distributed throughout much of the world and can have a significantly negative impact on sheep and goat industries [10,12,15,16]. In the U.S. alone, 36% of sheep operations contain SRLV infected sheep that result in decreased ewe and lamb productivity [15,17]. However, SRLV prevalence can be reduced through programs that incorporate aggressive testing and culling of seropositive sheep within flocks and repopulation with SRLV-free animals, and through the separation of lambs from seropositive dams immediately after birth with subsequent isolation from infected flocks [12,16].

Recently, a major host genetic component to sheep SLRV susceptibility was identified in the ovine transmembrane 154 gene (*TMEM154*) [18]. Ovine TMEM154 protein has a predicted signal peptide at its N terminus, as well as extracellular, intracellular, and transmembrane domains [18]. The function of this protein in sheep and other species is unknown, as is its biological role(s) in SRLV infection. Three *TMEM154* haplotypes that all encode for a full-length protein are commonly found in U.S. sheep [18]. Two haplotypes have a polymorphism allele that encodes a glutamate (E) amino acid within the extracellular domain of the protein (E35) (haplotypes #2 and #3, Table 1), whereas the other encodes a lysine (K) allele (K35, haplotype #1, Table 1). Both case–control and cohort studies have shown that sheep with a copy of either haplotype #2 or #3 have an increased risk of SRLV infection in comparison to sheep that are homozygous for haplotype #1 [18]. Consequently, the K35 allele shows potential as a genetic tool for the reduction of SRLV prevalence in sheep, and could be incorporated into SRLV control programs.

**Table 1:** ***TMEM154 *haplotype frequencies for 183 sheep.**

**Haplotype**	**Frequency**	**cgggg-[C,-]-gcgcg**	**cgccc-[T,A]-tttcc**	**tccca-[C,T]-ccgcc**	**aggag-[G,A]-acaca**	**acaca-[G,A]-aactg**	**aggca-[C,T]-ggaag**	**tataa-[A,T]-ttcta**	**accag-[TTAGAGTTTA,TTA]-tatta**
		4^a^	14	25	33	35	44	70	82
1	0.57	R	L	T	D	K	T	N	E
2	0.20	R	L	T	D	E	T	I	E
3	0.22	R	L	T	D	E	T	N	E
4	0.01	A^∆^	NA^b^	NA	NA	NA	NA	NA	NA

^a^Numbers refer to amino acid positions in reference sequence HM355886.

^b^Predicted non-functional isomer due to a deletion allele at amino acid position 4.

Regarding the genetics of host-pathogen interactions, it is important to account for variation in both pathogen and host populations, and thus the context in which either host or pathogen alleles associate with infection or disease. This is particularly relevant for ovine *TMEM154*-derived reduced SRLV susceptibility, as lentiviruses evolve at an accelerated rate, and some SRLV genetic subtypes appear to be geographically stratified throughout many locations of the world, including the United States [3,19-23]. Accordingly, the goals of this study were to 1) develop a phylotyping system for SRLVs in the U.S. based on proviral *gag* and *env* genomic variation, 2) phylotype SRLVs infecting sheep from the same U.S. location in which the *TMEM154* haplotype associations were first identified, and 3) test SRLV phylotypes for associations with *TMEM154* genotypes. We identified two SRLV genetic subgroups that are infecting U.S. sheep and that are distinct from SRLVs of European origins, and report that sheep with hemizygous or homozygous *TMEM154* K35 genotypes have an increased risk of infection by SRLVs of subgroup 1, as do sheep with hemi-or homozygous *TMEM154* E35 genotypes by SRLVs of subgroup 2. These results indicate that both host and SRLV genotypes affect the relative risk of SRLV infection in sheep, and that the success of SRLV control measures that incorporate the *TMEM154* K35 haplotype could be impacted by the types of SRLV strains present in endemically infected flocks.

## Materials and methods

### Animal cohorts used in study

Animal use was approved by the animal care and use committee of the United States Department of Agriculture, Agricultural Research Service, U.S. Meat Animal Research Center. Three animal cohorts at the U.S. Meat Animal Research Center in Nebraska were used for association testing of SRLV phylotypes with *TMEM154* E35K genotypes. One consisted of 57 SRLV seropositive sheep that were diagnosed with clinical ovine progressive pneumonia (OPP) through gross morphology and histopathology of both lung and mediastinal lymph node. The animals comprising this group consisted of four rams and fifty-three ewes that were born from 1998 to 2004 and had germplasm from Columbia, Dorset, Finn, Hampshire, Rambouillet, Romanov, Suffolk, and Texel breeds. Another cohort consisted of 97 non-clinical SRLV seropositive ewes born from 1994 to 1998 that had germplasm from Columbia, Dorset, Finn, Hampshire, Rambouillet, Romanov, and Suffolk breeds. The third cohort consisted of 29 non-clinical SRLV seropositive ewes that were born in 2005 and 2006 and were Rambouillet-Romanov reciprocal crossbreds. As reported in a previous study [18], all 183 animals within the three cohorts tested positive for SRLV infection via a competitive enzyme-linked immunosorbent assay (cELISA), (VMRD, Inc, Pullman, WA, USA) [24].

### Ovine *TMEM154* amplification and polymorphism genotyping

Two segments of *TMEM154* spanning exon 1, and two other segments spanning exon 2 were previously amplified, sequenced, and scored for the animals used in this study [18]. Eight polymorphisms observed in U.S. sheep reside on *TMEM154* exons 1 and 2. Exon 1 contains R4A^Δ^ which is a cytosine deletion (cgggg-[C,-]-gcgcg), L14H (cgccc-[T,A]-tttcc), and T25I (tccca-[C,T]-ccgcc). Exon 2 contains D33N (aggag-[G,A]-acaca), E35K (acaca-[G,A]-aactg), T44M (aggca-[C,T]-ggaag), N70I (tataa-[A,T]-ttcta), and E82Y^∆^ which is a seven base pair InDel involving *TMEM154* codons 81–83 (accag-[TTAGAGTTTA, TTA]-tatta). The eight polymorphism genotypes used in this study, in addition to others located throughout *TMEM154*, are annotated in GenBank file HM355886.2 [18,25].

### SRLV proviral *gag* amplification, cloning, and sequencing

**Figure 1:**
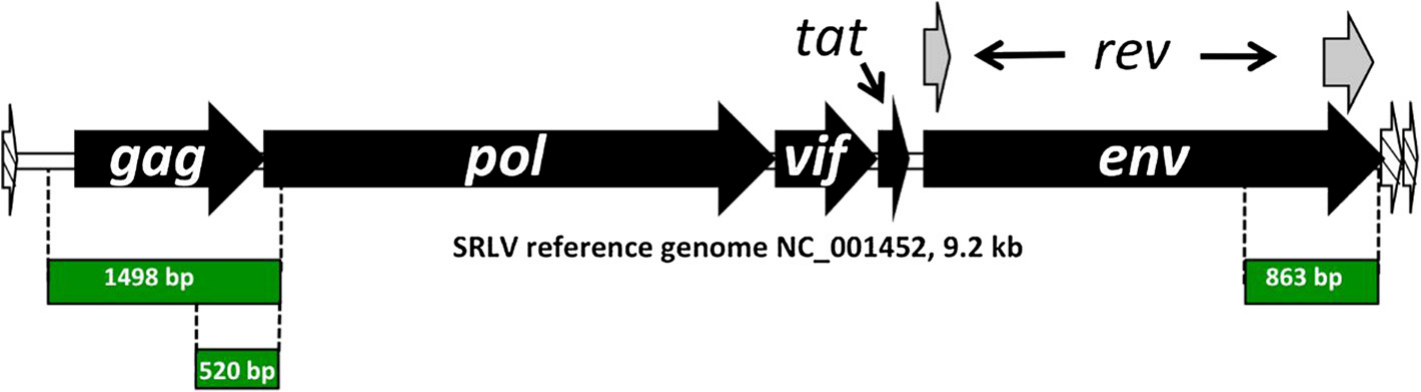
**Physical map of SRLV genome.** The white rectangle represents SRLV intergeneic sequence. Black block arrows represent genes. Grey block arrows represent exons. Hatched arrows represent long terminal repeats. Amplification products for *gag* (first and second round), and *env* (single round) are represented by green rectangles.

Figure 1 shows a physical map of a reference SRLV genome, and the *gag* and *env* regions targeted for proviral amplification, cloning, and sequencing. Proviral SRLV *gag* sequence was amplified from the blood-isolated DNAs of 126 SRLV infected, non-clinical sheep and the lung-isolated DNAs of 57 SRLV infected sheep with clinical OPP via nested PCR. Three of the four primers used for *gag* amplification were designed for this study from alignments of SRLV *gag* sequences available in GenBank. First round PCRs consisted of 0.4 μM of forward primer 83014 (5′-GGTAAGAGAGACACCTACTGG-3′) and a previously designed reverse primer *GAG*PSr (5′-GCGGACGGCACCACACG-3′) [26], which hybridized to a conserved region of *gag* in our alignment of GenBank sequences. Additionally, first round PCRs consisted of 1 ng/μL of DNA, 2 mM MgCl_2_, and 1X Thermo-start Taq DNA polymerase and buffer (ABgene, United Kingdom), and a total volume of either 27.5 or 55 μL. The PCR thermocycling conditions consisted of a 15 min incubation at 94°C, 40 cycles of: 94°C for 20 s, 60°C for 30 s, and 72°C for 1 min, and a final incubation at 72°C for 3 min. First round PCRs typically yielded an amplicon of approximately 1498 base pairs that was not visible by agarose gel electrophoresis (Figure 1). Second round PCRs consisted of 1.8% volume of the completed first PCR reaction, 0.4 μM of forward primer 87074 (5′-TATGYTTRCAATGGGTRATA-3′) and reverse primer 84156 (5′-ACACGTGGCCCCCTCCTG-3′), either 2 or 3 mM MgCl_2_, and 1X Thermo-start Taq DNA polymerase and buffer (ABgene), and a total volume of either 27.5 or 55 μL. The reactions yielded amplicons of approximately 520 base pairs (Figure 1). The second round thermocycling conditions were the same as the first round PCR; however, the number of cycles conducted for subsequent cloning and sequencing analyses was determined empirically for each sample so that a minimum number of cycles (25–40) was used to generate an amplicon for subsequent cloning.

Proviral *gag* amplicons were detected by 2% agarose gel electrophoresis, cloned into the pCRII® vector and electroporated into TOP10 cells per the manufacturer’s instructions (Invitrogen Life Technologies, Grand Island, NY, USA). Transformed cells were grown overnight on Lysogeny Broth Agar plates containing either kanamycin or amplicillin and Bluo-Gal (Invitrogen Life Technologies). Resultant colonies were screened for insert size via PCR using vector-specific M13 forward and reverse amplification primers (Invitrogen Life Technologies). Reactions yielding anticipated amplicon insert sizes were digested with Exonuclease I as previously described [27] and sequenced on an ABI 3730 capillary sequencer (PE Applied Biosystems, Foster City, CA, USA). Oligonucleotides M13 forward, M13 reverse, 87074, and 84156 were used as sequencing primers to obtain redundant amplicon sequence coverage. One to five clones were selected and sequenced for each *gag* amplification reaction.

### SRLV proviral *env* amplification, cloning, and sequencing

SRLV proviral transmembrane *env* sequence was amplified from the lung DNAs of 57 SRLV clinically ill sheep via one round of PCR. All of the primers used for *env* amplification and/or sequencing were designed from alignments of SRLV *env* sequences available in GenBank. The PCR consisted of 1 ng/μL of DNA, an equimolar 0.4 μM mix of forward primers 85938 (5′-GTCGTGCAGCAATCCTAYAC-3′), 85940 (5′-GCCGTGCAGCARTCCTAYAC-3′), and 85942 (5′-GCGATTCAGCAGTCTTAYAC-3′), and 0.4 μM of reverse primer 83639 (5′-CCTGACAGTCCACCCTTTC-3′), 1.5 mM MgCl_2_, 4% DMSO, and 1X Thermo-start Taq DNA polymerase and buffer (ABgene). The reaction volumes were typically either 27.5 or 55 μL. The PCR thermocycling conditions consisted of a 15 min incubation at 94°C, 30–40 cycles of: 94°C for 20 s, 58°C for 30 s, and 72°C for 1 min, and a final incubation at 72°C for 3 min. For each sample, a minimal number of amplifications was determined to produce an amplicon of approximately 863 base pairs for subsequent cloning and sequencing (Figure 1). Proviral *env* amplicons were purified with QIA quick PCR purification spin columns (Qiagen, Valencia, CA, USA) and subsequently cloned and processed for ABI 3730 sequencing as described above for *gag* amplicons. Oligonucleotides used as sequencing primers for *env* included M13 forward and M13 reverse, as well as forward primers 86814 (5′- AAGGGATAAGAATTTTAGAAGC -3′, 86815 (5′- TCTCATGGTTAAAGTATATCCC-3′), and 86817 (5′- ATATGTTTTAGAATTTTAATGTGTT-3′), and reverse pri-mers 86816 (5′- GGGATATACTTTAACCATGAGA -3′), 86818 (5′-AACACATTAAAATTCTAAAACATAT-3′), and 86820 (5′- TTTCCATTTGTGTCCCCA-3′). One to eighteen clones were selected and sequenced for each *env* amplification reaction.

### Assembly of SRLV *gag* and *env* clone sequences

Individual sequences generated for *gag* and *env* clones were assembled into consensus clone sequences using phred and phrap [28,29], polyphred (version 6.10) [30], and consed software [31]. Each consensus clone sequence was manually checked for amplicon coverage and sequence integrity. Vector and primer sequences were removed from each clone consensus sequence. Clones with sequences displaying heterozygous polymorphism alleles were the probable result of mixed colonies from the cloning steps and were excluded from the study.

### Phylogenetic and recombination analyses of *gag* and *env* sequences

The DNA alignments produced in this study were created in MacVector (version 12.0.6) using ClustalW. Mid-point rooted Neighbor-Joining trees were made in PHYLIP (version 3.69) [32] from alignments of 1) all *gag* sequences generated in this study, 2) 21 *gag* sequences that represented all 21 major *gag* phylotypes identified from the sequences generated in this study (Additional file 1) 3) 16 *gag* sequences that represented 16 of the 21 major *gag* phylotypes (Additional file 1), 4) all *env* sequences generated in this study, and 5) 16 *env* sequences that represented all 16 major *env* phylotypes identified from the sequences generated in this study (Additional file 1). The trees were created using PHYLIP programs DNADIST, NEIGHBOR, and RETREE, with an F84 model of substitution and a transition/transversion ratio of 2. Bootstrap values were calculated for the trees with 1000 pseudoalignments, distance matrices, and Neighbor-Joining trees, respectively, that were generated with SEQBOOT, DNADIST, and NEIGHBOR. A consensus tree was generated using CONSENSE. The trees were viewed with either DENDROSCOPE (version 3.1.0) [33], or Treeview (version 1.6.6) [34].

Select sequences collectively representing all 21 major *gag* phylotypes were tested for recombination using the Recombinant Identification Program (RIP), (Additional file 2) [35]. The test was conducted with homologous *gag* sequences from GenBank files AY101611 and GQ255430.1, with a window of 100 bases, allowance for multistate characters, and a 95% confidence interval. GenBank files AY101611 and GQ255430.1 both represent SRLVs of U.S. origin.

Neighbor-Net phylogenetic networks were made to account for recombinant sequences [36], and to compare the SRLV phylotypes identified in this study with others from around the world. Networks were generated in SplitsTree (version 4.12.3) [37] for alignments of 1) 21 sequences that represented all 21 major *gag* phylotypes (Additional file 1), 2) 16 sequences that represented the 16 major *env* phylotypes (Additional file 1), 3) 41 *gag* sequences that represented all 21 major *gag*-based phylotypes, along with 35 SRLV *gag* sequences available in GenBank (Additional file 3), and 4) 16 *env* sequences that represented all 16 major phylotypes along with 24 SRLV *env* sequences available in GenBank (Additional file 3). The Neighbor-Net networks were generated with an F84 model of substitution with a transition/transversion ratio of two.

### Statistical testing

Two SRLV subgroups defined by *gag* sequence variation were tested for associations with *TMEM154* E35K genotypes, as were two subgroups defined by *env* sequence variation, using 2-way contingency table analyses. The *gag* subgroups incorporated proviral sequences originating from 183 animals that were also haplotyped for *TMEM154* E35K. The *env* subgroups incorporated proviral sequences from 57 SRLV clinical cases, which were part of the 183 animals. For both *gag* and *env* subgroups, the association testing was conducted two ways, either with all observed *TMEM154* E35K genotypes (including *TMEM154* E35K heterozygotes), or with only hemi-, and homozygous *TMEM154* E35 and K35 genotypes, respectively (Additional file 4). Importantly, for both *gag* and *env* datasets, subgroup members were represented just once per animal for the association testing. If members of both subgroups were observed from an animal, both were represented once in the test.

### Nucleotide sequence accession numbers

All SRLV nucleotide sequences have been deposited in GenBank (*gag:* KF011980-KF012634, *env*: KF024749-KF025197). Each file is annotated for host animal breed, *TMEM154* E35K genotype, *TMEM154* diplotype, the tissue type used for DNA extraction and proviral amplification, and for any predicted premature stop codons in protein coding sequence.

## Results and discussion

### *TMEM154* haplotype and E35K frequencies

Four *TMEM154* haplotypes were found among the 183 sheep used in this study. The frequency of haplotype #1, which encodes K35 was 0.57 (Table 1). The frequencies of haplotypes #2 and #3, which both encode E35, were 0.20 and 0.22, respectively. Haplotype #4, which encodes a predicted non-functional protein isoform, was also observed but at a low frequency (0.01). Thirty-six percent of all 183 sheep were hemi- or homozygous for K35 (as a result of *TMEM154* 1,1 or 1,4 diplotypes (Table 2)). These sheep were grouped together for association testing with SRLV subgroups. Sixty-four percent of the sheep had one or more copy of either haplotype #2 or #3, and comprised an E35 group for association testing. Additionally, twenty percent of the sheep were hemi- or homozygous for E35 (as a result of *TMEM154* 2,2; 2,3; 2,4; or 3,3 diplotypes. These sheep were also grouped together for association testing. Thus, all *TMEM154* diplotypes were used for testing.

**Table 2:** ***TMEM154 *diplotype and E35K frequencies for all 183 sheep.**

**Diplotype**^**a**^	**1,1**	**1,2**	**1,3**	**1,4**	**2,2**	**2,3**	**2,4**	**3,3**
E35K Genotype	K,K	K,E	K,E	K,deletion^b^	E,E	E,E	E,deletion	E,E
Frequency	0.35	0.27	0.17	0.01	0.05	0.02	0.01	0.12

^a^Combinations of haplotypes defined in Table 1.

^b^Haplotype four codes for a predicted non-functional protein due to a deletion mutation.

### Identification of *gag* and *env* subgroups and their associations with *TMEM154* E35K genotypes

**Figure 2:**
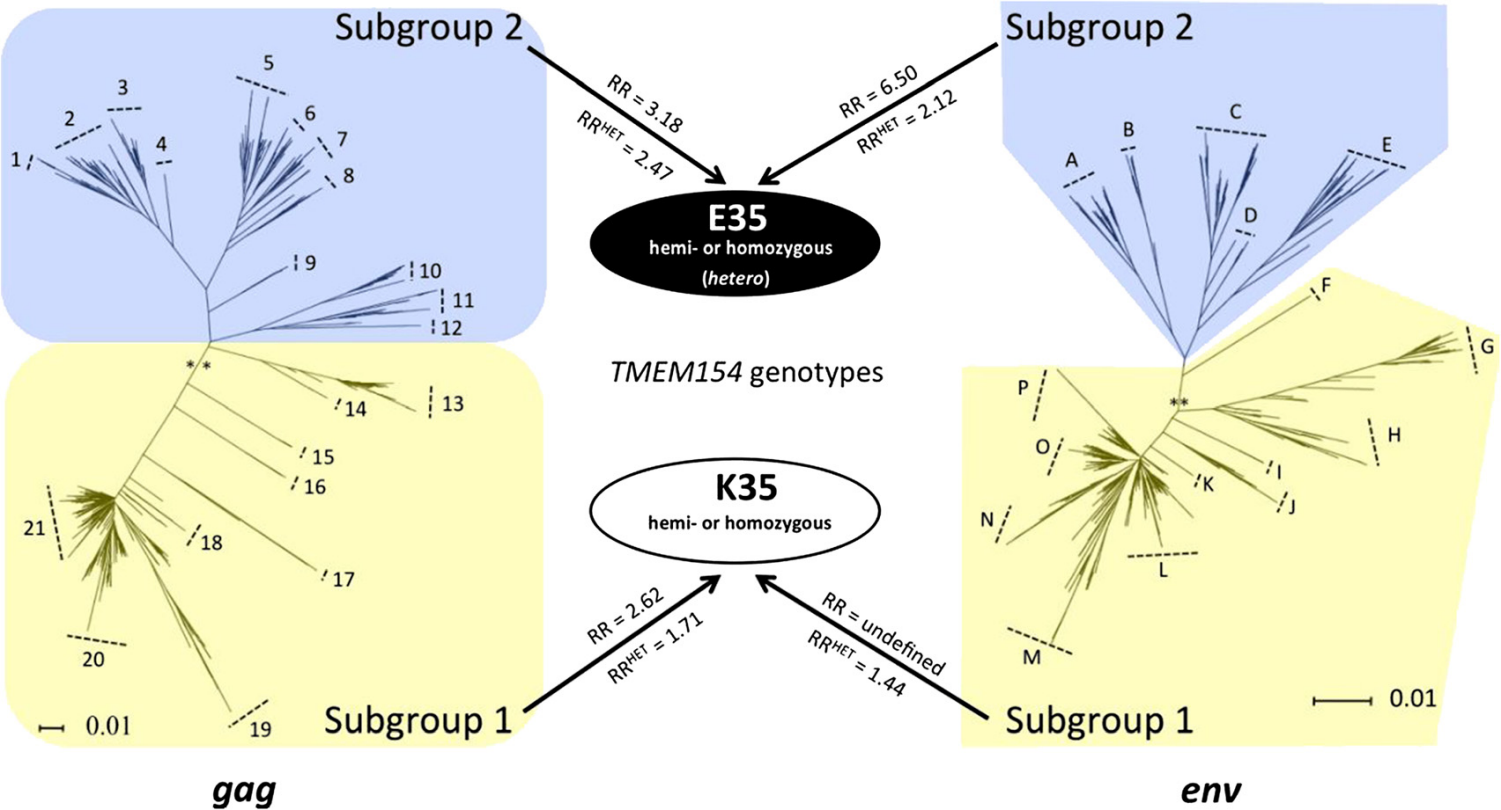
**Identification of SRLV *gag *and *env *subgroups that associate with *TMEM154 *E35K genotypes.** The *gag* and *env* Neighbor-Joining trees represent all 655 *gag* and 449 *env* sequences produced in this study, respectively. Numbers and letters represent phylotype designations in the *gag* and *env* trees, respectively. Double asterisks show the mid-point root site of the trees. Scale bars represent substitutions per site. Yellow and blue shaded regions represent subgroups 1 and 2, respectively, for both trees. Arrows show the relative risks of *TMEM154* E35 and K35 sheep infection by SRLV subgroups. RR^HETs^ were derived with grouped hemi-, hetero-, and homozygous *TMEM154* E35 sheep; and grouped hemi-and homozygous K35 sheep. RRs were derived with hemi and homozygous groups of *TMEM154* E35 sheep and K35 sheep, respectively. All hemizygous genotypes consisted of one copy of haplotype 4, which contained a deletion mutation and did not code for a predicted full-length biologically active isomer.

A two-step nested *gag* PCR assay was developed to identify SRLVs infecting sheep within the United States. The assay was used to generate a total of 655 *gag* sequences from the 183 SRLV seropositive sheep with known *TMEM154* E35K genotypes. Of the sequences, 467 coded for uninterrupted protein and 188 contained predicted premature stop codons. Between one to five *gag* sequences were generated for each animal. A Neighbor-Joining tree placed the sequences into 21 discrete clusters or phylotypes and was divided by midpoint rooting into two genetic subgroups (subgroups 1 and 2, Figure 2). Representatives of both SRLV subgroups were observed in all breeds used in this study with the exception of Finn sheep (*n* = 14 animals, data not shown).

A 2-way contingency table analysis that incorporated all *TMEM154* E35K genotypes showed that SRLV subgroup 1 associated with hemi- and homozygous *TMEM154* K35 genotypes and subgroup 2 associated with hemi-, heterozygous, and homozygous *TMEM154* E35 genotypes (Pearson Uncorrected Chi-square (χ^2^) = 14.7, *p* < 0.001). However, based on a stronger chi-square value (χ^2^ = 18.8, *p* < 0.001), the division for the two subgroups was changed to reclassify a branch of the tree comprised of three distinct phylotypes that was located very close to the midpoint root (Figure 2). Additionally, given that the distributions of heterozygous *TMEM154* E35K genotypes were proportional between the subgroups (subgroup 1, N = 42, subgroup 2, N = 34), the subgroup associations were retested using only hemi-and homozygous *TMEM154* K35 and E35 genotypes (χ^2^ = 26.5, *p* < 0.001), (Additional file 4). Sheep hemi- or homozygous for the K35 allele had a relative risk of 2.62 for infection by subgroup 1 SRLVs (95% confidence interval (95% CI) = 1.69-4.17), and sheep hemi-or homozygous for the E35 allele had a relative risk of 3.18 for infection by subgroup 2 SRLVs (95% CI = 1.95-5.15), (Figure 2, Additional file 4). Taken together, these results indicated that SRLV subgroups 1 and 2 have adapted to infect sheep with the *TMEM154* K35 and E35 allele, respectively, and that E35K heterozygous sheep are susceptible to infection by either subgroup.

In addition to *gag*, a PCR assay was developed to identify *env* SRLV subgroups infecting U.S. sheep, and to test them for an association with *TMEM154* genotypes. This second assay targeted the transmembrane region of *env* and allowed us to test an entirely different region of the SRLV genome for an association with *TMEM154* E35K genotypes, and to address potential resampling biases that could have impacted the *gag* assay results [38]. A total of 449 sequences was generated from a cohort of 57 sheep with clinical OPP. Of the *env* sequences, 422 coded for uninterrupted protein and 27 contained predicted premature stop codons. One to eighteen sequences were generated per animal. A Neighbor-Joining tree placed the sequences into 16 discrete phylotypes (Figure 2). Similar to the *gag* tree, the *env* tree was originally midpoint rooted to define two subgroups that each contained related *env* phylotypes, and the cutoff between the two subgroups was moved to reclassify the branch representative of a single sequence that placed very close to midpoint root.

A 2-way contingency table analysis that incorporated all *TMEM154* genotypes showed that *env* SRLV subgroup 1 associated with hemi- and homozygous *TMEM154* K35 genotypes and subgroup 2 associated with hemi-, heterozygous, and homozygous *TMEM154* E35 genotypes (χ^2^ = 3.9, *p* = 0.049), (Additional file 4). However, like the *gag* subgroups, the distributions of heterozygous *TMEM154* E35K genotypes were proportional between the *env* subgroups (Subgroup 1, N = 18 Subgroup 2, N = 12), thus the subgroup associations were retested using only hemi-and homozygous *TMEM154* E35 and K35 genotypes (χ^2^ = 10.5, *p* = 0.01). Because sheep hemi-or homozygous for the E35 allele were not found infected with subgroup 1 SRLVs, the relative risk of sheep hemi- or homozygous for the K35 allele being infected by subgroup 1 SRLVs could not be defined, and sheep hemi- or homozygous for the E35 allele had a relative risk of 6.5, which matched the upper 95% CI limit (95% CI=1.45-6.5), (Figure 2, Additional file 4). Thus, both *gag* and *env* sequence variants defined two SRLV subgroups with similar associations with *TMEM154* E35K genotypes.

### Comparison of *gag* and *env* subgroups

SRLV *gag* and *env* subgroup assignments were compared within the cohort of 57 sheep with clinical OPP using all *TMEM154* E35K genotypes. Fifty-two sheep yielded sequences that exactly matched by their *env* and *gag* subgroup assignments. This included one animal that yielded sequences that placed in subgroups 1 and 2 of both *gag* and *env,* indicating it may have been infected by SRLVs more than once. Two sheep had SRLV sequences that placed in both *env* subgroups and one *gag* subgroup, and one animal had SRLV sequences that placed in both *gag* subgroups and one *env* subgroup. Only two of the 57 sheep had a direct conflict of SRLV *gag* and *env* subgroup assignments, with both yielding sequences that placed in *env* subgroup 2 and *gag* subgroup 1. Consequently, the *gag* and *env* subgroups corresponded in their associations with *TMEM154* E35K genotypes.

### Phylogenetic stability of *gag* and *env* subgroups

**Figure 3:**
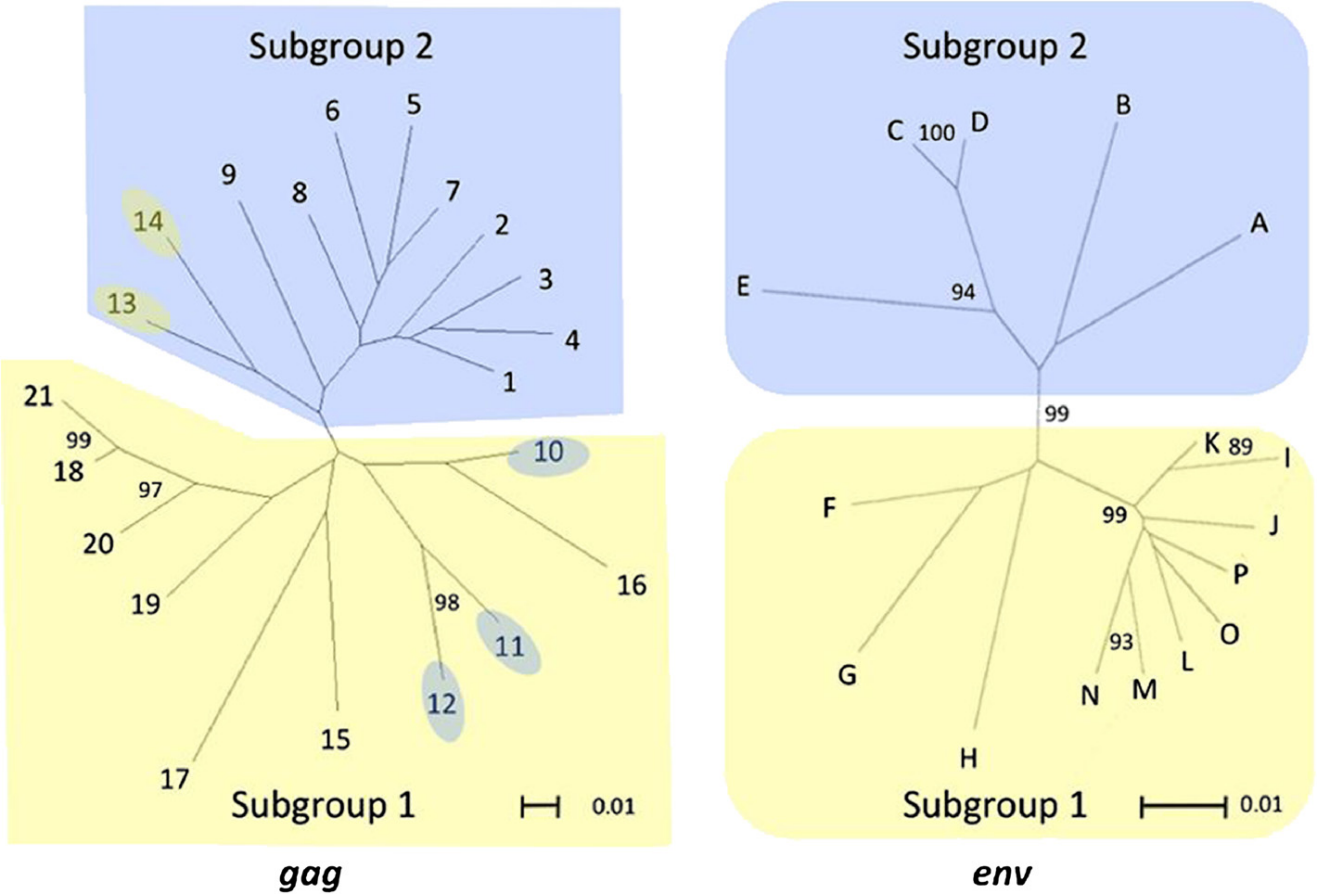
**Phylogenetic stability of all *gag *and *env *phylotypes within subgroups.** The *gag* Neighbor-Joining tree contains sequences representing the 21 *gag* phylotypes identified in Figure 2. The *env* Neighbor-Joining tree contains sequences representing the 16 *env* phylotypes identified in Figure 2. Numbers and letters located at outer taxonomic units represent phylotype designations in the *gag* and *env* trees, respectively. Numbers at internal nodes of the trees represent bootstrap values. Yellow and blue shaded regions represent subgroups 1 and 2, respectively, for both trees. Conflicting subgroup placement for gag phylotypes 10–14 relative to Figure 2 are represented with colored ovals. Scale bars represent substitutions per site.

**Figure 4:**
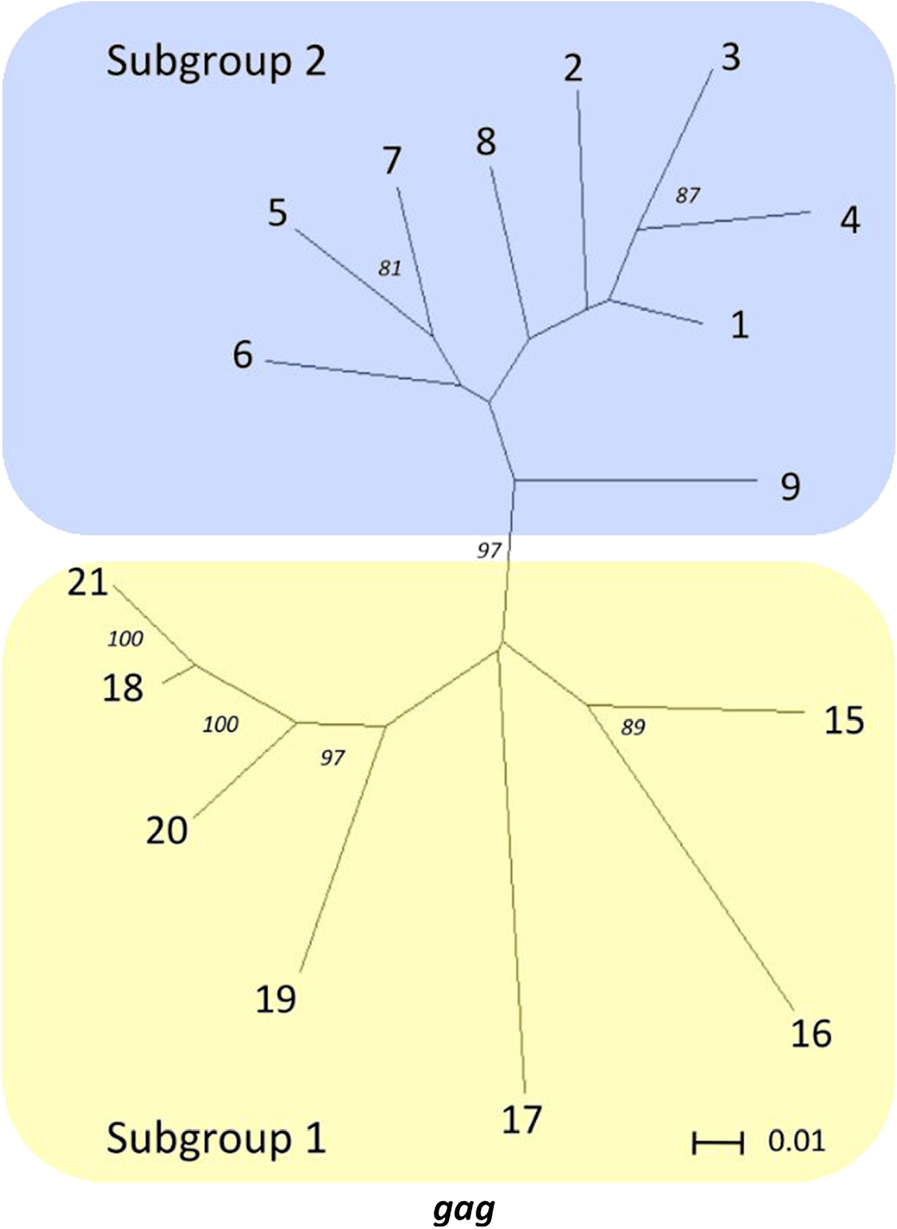
**Phylogenetic stability of *gag *subgroups without phylotypes 10–14.** The *gag* Neighbor-Joining tree contains sequences representing 16 of 21 *gag* phylotypes identified in Figure 2. Numbers located at outer taxonomic units represent phylotype designations. Numbers at internal nodes of the trees represent bootstrap values. Yellow and blue shaded regions represent subgroups 1 and 2, respectively. The scale bar represents substitutions per site.

The phylogenetic stability of *gag* and *env* subgroups and phylotypes was assessed with bootstraps using Neighbor-Joining trees of representative sequences for the 21 *gag* and 16 *env* phylotypes (Figure 3). Separation of subgroups 1 and 2 was strongly supported by all 16 *env* phylotypes with a bootstrap value of 99%. Similarly, separation of subgroups 1 and 2 was strongly supported by 16 of 21 *gag* phylotypes with a bootstrap of 97% (Figure 4). However, five low frequency phylotypes (10–14) did not support subgroup 1 and 2 separation with high bootstrap values, and were unstable in their subgroup placements (Figure 3). Given that phylotypes 10–14 placed very close to the mid-point roots of the Neighbor-Joining trees of Figures 2 and 3, they did not clearly delineate the phylogenetic boundaries of subgroups 1 and 2.

**Figure 5:**
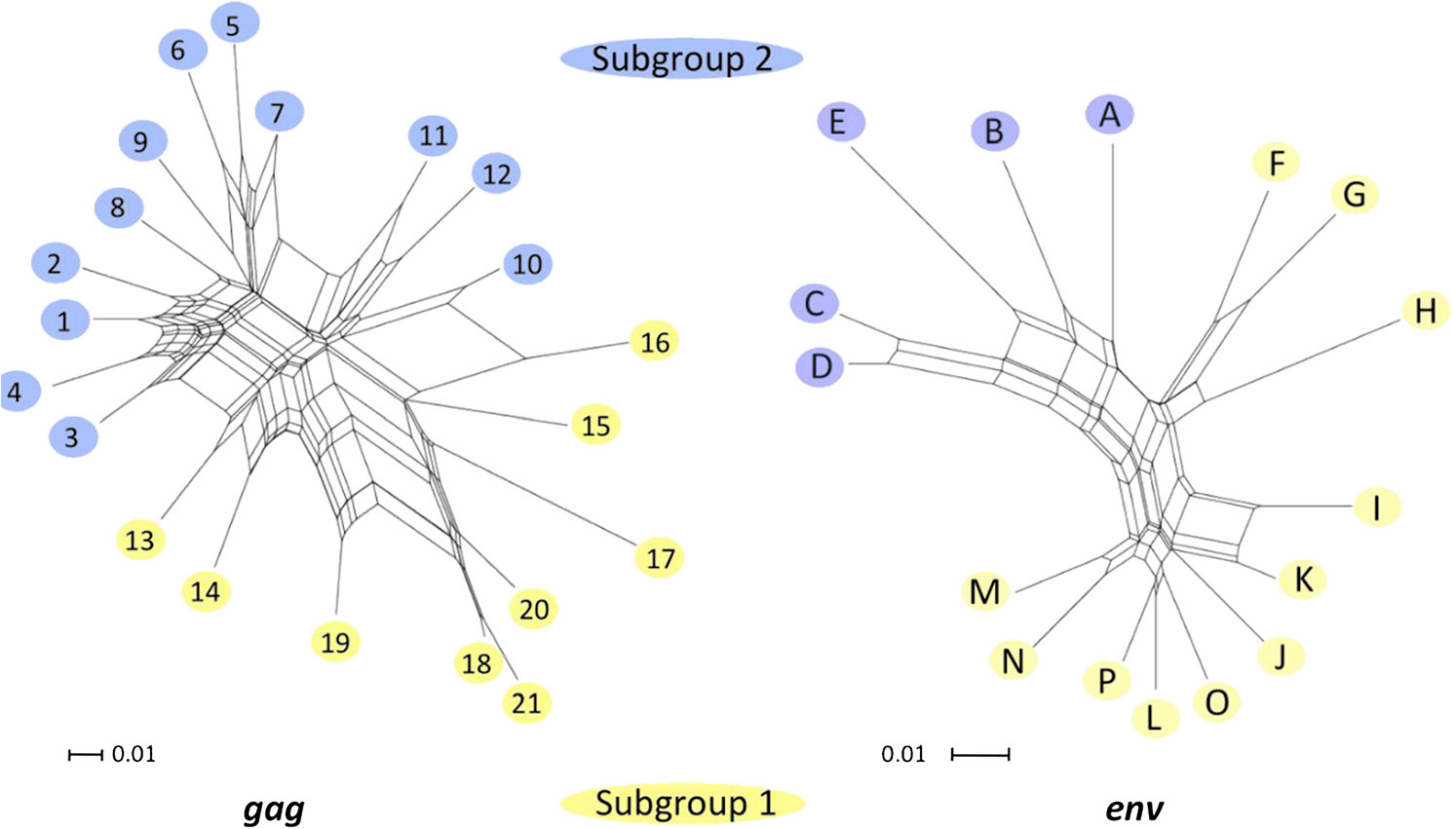
***gag *and *env *Neighbor-Joining networks.** The *gag* network contains sequences representing the 21 *gag* phylotpes identified in Figure 2. The *env* network contains sequences representing the 16 *env* phylotypes identified in Figure 2. Scale bars represent substitutions per site.

Low bootstrap values and/or unstable placements in Neighbor-Joining trees can be an indicator of recombination, and Neighbor-Joining trees are not particularly effective in depicting accurate phylogenetic reconstructions of recombinant sequences [39,40]. Given that recombinant sequences could have complicated some of our phylogenetic analyses, representative sequences from each of the 21 *gag* phylotypes identified in this study were tested for recombination. Twelve of the 21 phylotypes tested positive for recombination with homologous SRLV *gag* sequence from GenBank that represented subgroup 1 (AY101611) and subgroup 2 (GQ255430.1) at a 95% confidence interval (Additional file 2). Accordingly, independent Neighbor-Net phylogenetic networks were constructed from representative sequences of 1) all 21 *gag* phylotypes, and 2) all 16 *env* phylotypes identified in this study, as the networks accounted for recombination (Figure 5). Both networks were split into two subgroups. The phylotype composition of the two subgroups within *env* Neighbor-Joining trees and networks were identical (Figures 2, 3, and 5). Additionally, the networks placed *gag* phylotypes 1–12 in subgroup 2 and phylotypes 13–21 in subgroup 1, a result identical with the Neighbor-Joining tree representing all 655 *gag* sequences generated in this study (Figures 2 and 5). Thus, while identification of the exact phylogenetic boundary separating the *gag* subgroups will require additional research, these results indicate that the SRLV subtype associations with *TMEM154* E35K genotypes were not artifacts of recombinant sequences.

### Classification of *gag* phylotypes

**Figure 6:**
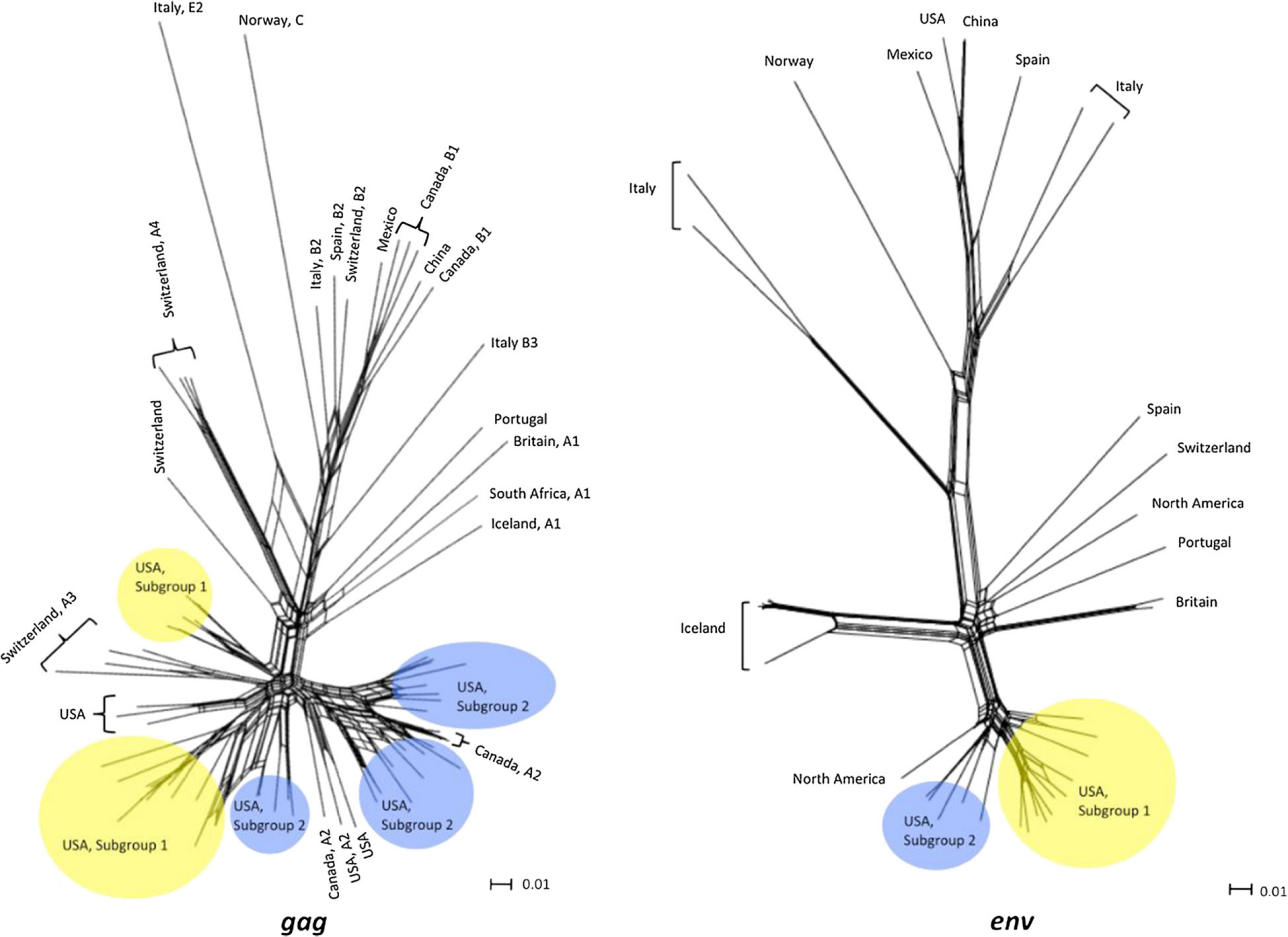
**Neighbor-Joining network comparisons of sequences produced in this study with those available from GenBank.** Sequences representing SRLVs of known geographical origin are labeled in the networks. Capital letters and numbers following the country of origin in the *gag* network represent previously reported genotypes and subtypes, respectively. The scale bars represent substitutions per site.

SRLVs have been previously classified into genotype groups A-E, of which some are further resolved into subtypes [22,23,41-43]. To determine the placement of *gag* phylotypes that varied in their association with *TMEM154* E35K genotypes in the established SRLV genotype group classification system, a Neighbor-Joining network was constructed from representative sequences of the 21 *gag* phylotypes characterized in this study and available SRLV *gag* sequences in GenBank with known genotype designations (Figure 6). Multiple unresolved loops in regions of the network that did not represent sequences from this study suggested that many available SRLV *gag* sequences in GenBank were either recombinant, or contained regions of convergent evolution. The network additionally showed that the phylotypes of subgroup 2, which associated with hemi- and homozygous *TMEM154* E35 genotypes, were very similar to SRLV genotype A2, a subtype harbored in SRLVs from the United States and Canada. Based on the network, some, if not all of the phylotypes that comprise subgroup 2 belong to the A2 genotype, and some members of the A2 genotype are not likely to infect sheep with a genetic barrier to infection associated with *TMEM154* K35.

Phylogenetic classification of the subgroup 1 phylotypes was less conclusive than subgroup 2. The Neighbor-Joining network showed that subgroup 1 phylotypes were similar to the *gag* sequences of SRLVs that originated from a northwest region of the United States, and to SRLV genotype A3 representatives that originated from Switzerland. SRLV *gag* subtypes from the U.S. and Switzerland have previously been shown to cluster together [26,42], indicating that they may share a recent common ancestor. However, both of the two cluster representatives of subgroup 1 were distinct from the A3 cluster in the network of Figure 6 and may represent new subtypes of the “A” Genotype. The distribution of subgroup 1 phylotypes in the network suggests that SRLVs with an increased propensity to infect sheep with hemi- and homozygous *TMEM154* K35 genotypes may be dispersed throughout several locations of the United States, and possibly Europe as well. However, estimates of the extent to which SRLV subtypes that originate from regions outside of North America may associate with *TMEM154* E35K genotypes are inherently problematic due to the clustering of subgroups 1 and 2 in one region of the network, and probable recombination or convergent evolution events that are represented across the network.

### Classification of *env* phylotypes

The SRLV proviral *env* sequences produced in this study were distinct from European *env* sequences available in GenBank (Figure 6). Both *env* subgroups 1 and 2 placed on one end of the Neighbor-Joining network of Figure 6 along with a North American SRLV sequence. Thus, as with the *gag* network, it is difficult to estimate how subtypes on the network that do not belong to subgroups 1 and 2 may associate with *TMEM154* E35K genotypes. The one *env* sequence on the network that represented a SRLV from Switzerland was an A4 subtype that did not cluster with *env* subgroups 1 and 2. Interestingly, North American *env* sequences from GenBank placed throughout several regions of the network, including close clustering with SRLV sequences that originated from Spain and Portugal, and additional clustering with an SRLV sequence that originated from China (Figure 6). These results emphasize the utility of *env* variation in detecting SRLV subtypes and possible strain migration patterns across geographical areas.

### Summary and future research

SRLVs have adapted to infect sheep with specific *TMEM154* E35K genotypes. Consequently, sheep with a genetic barrier to infection associated with homozygous *TMEM154* K35 genotypes are more likely to be infected by SRLVs of subgroup 1 versus members of subgroup 2. SRLVs of subgroup 1 are distributed in several regions of the United States and share some similarity to variants in Switzerland. However, while the results of this study show associations between SRLV subgroups and *TMEM154* E35K genotypes, additional research is necessary to unravel the genetics responsible for the associations. First, the boundary between both *env* and *gag* genetic subgroups needs to be better defined through additional sampling, and/or extended proviral genome sequencing. For both *gag* and *env* trees and networks, some phylotypes placed very close to the mid-point branches and the cutoffs for subgroups 1 and 2. This potentially allowed for misclassification of some phylotypes within subgroups, particularly *gag* phylotypes 10–14 (Figures 2 and 3). Relatively few animals in this study were infected with SRLVs that placed in phylotypes 10–14, thus misclassification of some, or all of phylotypes 10–14 would not have changed our association findings. However, SRLVs with phylotypes 10–14 may be more frequent in sheep populations outside of our study group, thus correct subgroup classification is important for all phyotypes, including those of low frequency in our sheep populations.

Additionally, neither *gag* nor the transmembrane region of *env* may contain alleles that are biologically causative for the association of SRLV subgroups with *TMEM154* E35K genotypes. Given that *gag* and *env* are typically located on opposite ends of lentivirus genomes, and that genetic variants in both genes showed an association with *TMEM154* E35K genotypes, there may be a substantial genetic element in the genome that is biologically responsible for the association and is linked to genetic variation in both *gag* and the transmembrane region of *env*. This element could be in the region of *env* homologous to the glycoprotein 120 gene of HIV, as this gene codes for a docking protein that extends out of the viral membrane and into the external milieu of the virus [1,44], or it may be elsewhere in the genome. While the causative viral genetic element remains to be identified, the results of this study indicate that both host and SRLV genotypes affect the relative risk of SRLV infection in sheep.

## Abbreviations

ARS: Agricultural research service; BD: Becton Dickinson; C: Celsius; CA: California; CAEV: Caprine arthritis encephalitis virus; CE: Ceará; cELISA: Competitive enzyme-linked immunosorbent assay; CI: Confidence interval; DMSO: Dimethyl sulfoxide; DNA: Deoxyribonucleic acid; E: Glutamate; EDTA: Ethylenediaminetetraacetic acid disodium; env: Envelope gene; gag: Group-specific antigen; HIV: Human immunodeficiency virus; HRP: Horseradish peroxidase; Inc: Incorporated; K: Lysine; K2: Dipotassium; NC: North Carolina; NE: Nebraska; OPP: Ovine progressive pneumonia; PCR: Polymerase chain reaction; PE: Perkin Elmer; RIP: Recombinant identification program; RR: Relative risk; SRLVs: Small Ruminant lentiviruses; TMEM154: Transmembrane 154 gene; TMEM154 E35K: Nucleotide polymorphism encoding either a glutamate or lysine amino acid at position 35 of the full length transmembrane 154 protein; U.S.: United States; USA: United States of America; USDA: United States Department of Agriculture; USMARC: United States Meat Animal Research Center; VMRD: Veterinary Medical Research & Development; WA: Washington; WY: Wyoming.

## Competing interests

The authors declare that they have no competing interests.

## Authors’ contributions

Conceived and designed the experiments; LHS, MPH, CGCM, GPH, TPLS, KAL, WWL, MLC. Performed the experiments: LHS. Contributed reagents/materials/ analysis tools: LHS, MPH, CGCM, GPH, TPLS, KAL, WWL, MLC. Analyzed the results: LHS, MPH, CGCM, GPH, TPLS, KAL, WWL, MLC. Wrote the manuscript: LHS and MLC. Revised the manuscript with critical intellectual content: LHS, MPH, CGCM, GPH, TPLS, KAL, WWL, MLC. All authors read and approved the final manuscript.

## Supplementary Material

Additional file 1 **Sequences used to construct the phylogenetic trees or networks of Figures **3**, **4**and **5**.** Clone names, GenBank numbers, sequence type (*gag* or *env*) and subcluster assignments are provided for 37 SRLV sequences used to construct the phylogenetic trees or networks of Figures 3, 4 and 5. Click here for file

Additional file 2 ***gag* sequences tested for recombination.** Clone names, GenBank numbers, subcluster assignments and recombination test results are provided for 21 sequences that represent all 21 *gag* phylotypes identified in this study. Click here for file

Additional file 3 **SRLV sequences used for networks in Figure **6**.** The following information is provided for 117 SRLV sequences used to generate the networks of Figure 6: Sequence type (*gag* or *env*), GenBank numbers, clone names, phylotype numbers, SRLV subtype numbers, country of origin, and whether or not the sequences were produced in this study. Click here for file

Additional file 4 **2-way contingency tables used to calculate *gag *and *env* SRLV subgroup associations with sheep *TMEM154* E35K genotypes, and the relative risks shown in Figure **2**.** Four 2-way contingency tables are provided that were used to calculate the relative risk of: 1) K35 hemizygous or homozygous sheep infection by subgroup 1 SRLVs using hemi, hetero, or homozygous E35 genotypes, 2) E35 hemizygous, homozygous, or heterozygous sheep infection by subgroup 2 SRLVs using hemi or homzygous K35 genotypes, 3) K35 hemizygous or homozygous sheep infection by subgroup 1 SRLVs using hemi or homozygous E35 genotypes, and 4) E35 hemizygous or homozygous sheep infection by subgroup 2 SRLVs using hemi or homozygous K35 genotypes. Click here for file
